# Prevalence of child marriage and associated factors among reproductive age women in Harari regional state, eastern Ethiopia, 2022: a community-based study

**DOI:** 10.1186/s12905-023-02409-w

**Published:** 2023-05-16

**Authors:** Magarsa Lami, Abraham Negash, Addis Eyeberu, Abdi Birhanu, Adera Debella, Tamirat Getachew, Bekelu Berhanu, Bikila Balis, Tilahun Bete, Tilahun Abdeta, Shambel Nigussie, Kasahun Bogale, Deribe Bekele Dechasa, Addisu Sertsu, Kabtamu Gemechu, Dawud Wodaje, Kabtamu Nigussie, Ayichew Alemu, Haregeweyn Kibret, Kefelegn Bayu, Fentahun Meseret, Yideg Abinew, Fenta Wondimneh, Gebisa Dirirsa, Abduro Gobena, Jemal Husen, Addisu Alemu, Yadeta Dessie

**Affiliations:** 1 School of Nursing and Midwifery, College of Health and Medical Sciences, Haramaya University, Harar, Ethiopia; 2 School of Medicine, College of Health and Medical Sciences, Haramaya University, Harar, Ethiopia; 3 School of Pharmacy, College of Health and Medical Sciences, Haramaya University, Harar, Ethiopia; 4 School of Laboratory, College of Health and Medical Sciences, Haramaya University, Harar, Ethiopia; 5 School of Environmental Health, College of Health and Medical Sciences, Haramaya University, Harar, Ethiopia; 6 School of Public Health, College of Health and Medical Sciences, Haramaya University, Harar, Ethiopia

**Keywords:** Child marriage, Reproductive age, Prevalence, Associated factors, Harari region, Ethiopia

## Abstract

**Introduction:**

Child marriage is a union before the age of 18 and a violation of human right. Around 21% of young women in the world married before reaching the age of 18. Every year, 10 million girls under the age of 18 are married. Child marriage causes lifetime suffering, and its abolition was one component of the Sustainable Development Goal to achieve gender equality and empower women and girls. However; abolition of child marriage by 2030 will not happen because its prevalence in the community has remained stable.

**Objective:**

To assess the prevalence of child marriage and its associated factors among reproductive-age women from March 7 to April 5, 2022 in Harari Regional State, eastern Ethiopia.

**Methods:**

Community-based cross-sectional study was conducted from March 7 to April 5, 2022 among the reproductive age group in the Harari Region state, Eastern Ethiopia. A systematic random sampling technique was used to find study participants. Data were obtained by face-to-face interview using a pre-tested structured questionnaire, input into EpiData version 3.1 and analyzed using Stata version 16. The proportion with 95% confidence interval (CI) and the summery measure were used to report the prevalence. A multivariable logistic regression analysis model was used to examine associated factors, and the results were provided as an adjusted odds ratio (AOR) with a 95% confidence interval.

**Result:**

In this study 986 were responded to the interview, making response rate of 99.6%. The median age of study participants was 22 years. The prevalence of child marriage was 33.7% [95% CI: 30.8–36.7] in this study. Being a Muslim (AOR = 2.30, 95% CI = 1.26, 4.19), diploma or higher level of education (AOR = 0.26, 95%CI = .10, 0.70), rural residence (AOR = 5.39, 95% CI = 3.71, 7.82), a marriage arranged by others (AOR = 2.68, 95% CI = 1.49, 4.82) and not knowing legal age of marriage (AOR = 4.49, 95% CI = 2.57, 7.85) were significantly associated with child marriage.

**Conclusion:**

According to this report, nearly one out of every three women engages in child marriage. The practice was more common among those with lower educational attainment, those who lived in rural areas, people who were unaware of the legal age of marriage, and those whose engagement was decided by others. Focusing on strategies that allow for intervention in these factors is beneficial in ending child marriage, which has a direct and indirect impact on women's health and educational achievement.

## Introduction

Marriage is a personal choice, even if birth, marriage, and death are the three most important events in most people's lives. The right to make that choice is recognized as a legal principle and has long been enshrined in international human rights treaties [1]. Many girls, and to a lesser extent, boys, marry without exercising their right to choose. Some young females are coerced to marry. Others are too young to make an informed choice about a marriage partner or the consequences of marriage. They may accept consent given on their behalf by others [1,2

Globally, approximately 21% of young women married before the age of eighteen. Every year, 10 million girls under the age of 18 are married [3]. Africa shares the largest proportion of child marriage in the globe, with 54.0% of women in Africa experiencing child marriage [4]. The world's highest rates of early child marriage were found in Sub-Saharan Africa. The Sub-Saharan area was home to 18 of the world's top 20 countries with the highest rates of child marriage. There is also evidence that more than half of the girls in sub-Saharan Africa marry before their 18^th^ birthday [5, 6]. It varies between countries and it is high in Chad (61%), Mozambique (48%), Nigeria (43%), Ethiopia (40%), and Tanzania (31%) [7].

Child marriage practice has become a major social concern in recent years due to potentially harmful health implications such as an increased chance of contracting sexually transmitted infections, child malnutrition, teen pregnancy, missed opportunities in education, school dropout, and mother and child morbidity and mortality [8, 9]. It can also result in long-term suffering and an increased risk of domestic violence [10]. Furthermore, women married at < 18 years had frequent pregnancy termination and a rapid repeat of childbirth [4]. Young teenage girls are more likely to die due to complications in pregnancy and childbirth than women in their 20 s, and their children are more likely to be stillborn or die in the first month of life [4].

For married women in Ethiopia, child marriage has major health and social effects. Negative pregnancy outcomes, missed opportunities for formal education, a lack of opportunities for salaried employees, and social power inequities such as sexual violence, unequal profit-producing opportunities, a lack of money for basic necessities, and gender inequality in and out of their households are just a few of the consequences [11].

According to EDHS 2016 (Ethiopian Demographic Health Survey), Ethiopia is the home to 15 million child brides, and also 10% of girls aged between 15 and 19 years old are already mothers and early pregnancy leads to low birth weight, school dropout, abuse, violence, exploitation, fistula and other health risks for both babies and mothers [12]. In Ethiopia, the largest reported prevalence of child marriage was 44.8%, and 48.7% [13, 14]. Early marriage is commonly reported in Tigray, Amhara, and Afar regions [15]. The prevalence of marriage for girls aged 10–14 and 15–18 was 15.2% and 32.3%, respectively, in East Hararge Zone, indicating that both proportions were the highest in Oromia region for both groups [16].

The Ethiopian government's national strategy was a community mobilization campaign to end child marriage by 2025. Eliminating child marriage was one of the Sustainable Development Goals for achieving gender equality and empowering women and girls [17], However, based on current trends, child marriage will not be eliminated by 2030 [18].

Despite the government's best efforts, and the interventions of different stakeholders, the prevalence and factors associated with child marriage continue to occur. Furthermore, to the best of the author's knowledge concerned, there is a paucity of research regarding predictors of child marriage among the reproductive age groups in the study area. Therefore, to assist those working on the problem in taking evidence-based coordinated measures this study aimed to assess the prevalence of child marriage and its associated factors in Harari region eastern Ethiopia.

## Methods and materials

### Study design, study setting, and study period

A community-based cross-sectional study design was conducted from March 7 to April 5, 2022. Harari Region is one of Ethiopia's twelve regional states. It is located 526 km southeast of Addis Ababa, the capital city of Ethiopia. It has a total population of 232,000 of whom 116,928 are males and 115,072 females. It has 9 woredas’ (3 rural and 6 urban) and 36 kebeles’ (17 rural and 19 urban) with total households of 59,487. It has an estimated area of 333.94 square kilometers, with an estimated density of 595.9 people per square. The region is bounded by Oromia regional state districts in all directions (Harari regional health bureau, personal communication).

### Population and eligibility criteria

A source population included all reproductive-age women in the Harari regional state. The study population consisted of all randomly selected reproductive age women residing in selected kebeles of Harari regional state from March 7 to April 5, 2022. Those who did not stay in designated kebeles for more than six months or who were critically ill were excluded from the study.

### Sample size and sampling procedures

We calculated the sample size by using single population proportion formula with the assumptions of: Z_α/2_ = 1.96, 95% confidence level, 4% margin of error, design effect = 1.5, 48.57% proportion of child marriage from a previous study conducted in the Amhara region, Northern Ethiopia [14]. The determined sample size was 990 after accounting for a 10% non-response rate contingency. The study participants were sampled using a multi-stage sampling procedure. The Harari region comprises nine woredas with a total of 36 kebeles, 13 of which were chosen using the simple random sampling technique. The numbering of households in selected kebele was done by data collectors and the total households were 17,928. Households were proportionally allocated for each selected kebeles. Each household was chosen by using a systematic random sampling technique with a k value of 18, with the first household being chosen by lottery, and then, the eligible individual from the chosen household was included in the study. When more than one eligible study subject was found in a single household, the lottery method was used to pick only one respondent. When no eligible respondents could be found in the first household, the data collectors moved on to the next. This method resulted in the selection of 990 study-eligible participants from households.

### Tool and procedure for data collection

The information was gathered through a face-to-face interview using a pre-tested structured questionnaire designed after evaluating literature [9, 14, 19]. The questionnaires included questions about socioeconomic and demographic data. After a five-day training on the instruments and survey procedures, ten Bachelor of Science (BSc) nurses collected the data.

### Study variables

#### Dependent variables

Child marriage was the outcome variable of this study.

**Independent variables** include socio-demographic factors such as age, gender, occupation, educational level, income, marital status, and place of residence; Cultural factors like cultural beliefs. Factors related to obstetrics and health facilities, such as pregnancy problems, health facility accessibility, and information exposure.

#### Operational definitions and measurement

**Child marriage** was measured as any marriage occurring before the age of 18 years, in which the girl is not ready for marriage and delivery [20, 21]. Cultural beliefs; will be beliefs that are learned and shared across groups of people related to child marriage, one of the following beliefs is considered: HIV/AIDS, virginity, tradition, or ignorance related [22, 23].

#### Data quality control

The questionnaire was prepared in English and then translated by a language expert into the local languages (Afaan Oromoo and Amharic). The tool was then re-translated into English to guarantee consistency. The data collectors and field supervisors received training on the data collection tool and procedures. The pretest was conducted in comparable settings with 10% of the participants before the main study data collection. Regular supervision was provided by the investigators and competent field research supervisors.

#### Data processing and analysis

The collected data were checked for completeness, cleaned, coded, and entered into EpiData version 3.1. Then, the data were exported to Stata version 16 for analysis. Descriptive and summary statistics were done and the information was presented using tables and figures.

A binary logistic regression model was fitted to check if there was a relationship between the independent factors and the outcome variable. Hosmer–Lemeshow statistics and Omnibus tests were used to assess the model's fitness. All variables with *p*-value < 0.25 in the bivariate analysis were included in the final multivariate analysis. The standard error and co-linearity statistics were used to test for the presence of correlation between independent variables in a multi-collinearity test. The odds ratio (OR) and the 95% confidence interval were used to determine the direction and strength of the statistical association.

## Results

### Socio-demographic characteristics

From a total of 990 study participants, 986 were responded to the interview, making response rate of 99.6%. The median age of the study participants was 22 years, with an interquartile range of 20- 25 years, and a range from 18 to 43 years. 793 (80.43%) of the study participants were married. Nearly half of the study participants 485 (49.19%) had no formal education, and 619 (62.78%) study participants were Muslim (Table 1).

**Table 1:** Socio-demographic characteristics of participants of reproductive-age women residing in selected kebeles of Harari regional state, eastern Ethiopia, 2022, (*n* = 986)

Variables	Frequency (%)	Percent (%)
Age of participant		
< 20	358	36.31
20–29	539	54.67
30–39	74	7.51
≥ 40	15	1.51
Marital status of participants		
Married	793	80.43
Divorced	121	12.27
Widowed	54	5.48
Separated	18	1.82
Religion		
Muslim	619	62.78
Orthodox	243	24.65
Protestant	86	8.72
^a^Others	38	3.85
The educational level of the participants		
Had no formal education	485	49.19
Primary	185	18.76
Secondary	185	18.76
Diploma and above	131	13.29
The educational level of the Husband		
Had no formal education	416	42.19
Primary	196	19.88
Secondary	198	20.08
Diploma and above	176	17.85
Occupation of women		
Housewife	544	55.17
Merchant	195	19.78
Government employee	108	10.95
^b^Others	139	14.10
Husband occupation		
Farmer	445	45.13
Merchant	256	25.96
Government employee	155	15.72
^b^Others	130	13.18
Residence of participant		
Urban	584	59.23
Rural	402	40.77

^a^Indicates that (Adventist, Catholic, and Wakefata)

^b^Indicates that (private work, unemployed and student),

### Marriage characteristics

**Fig. 1:**
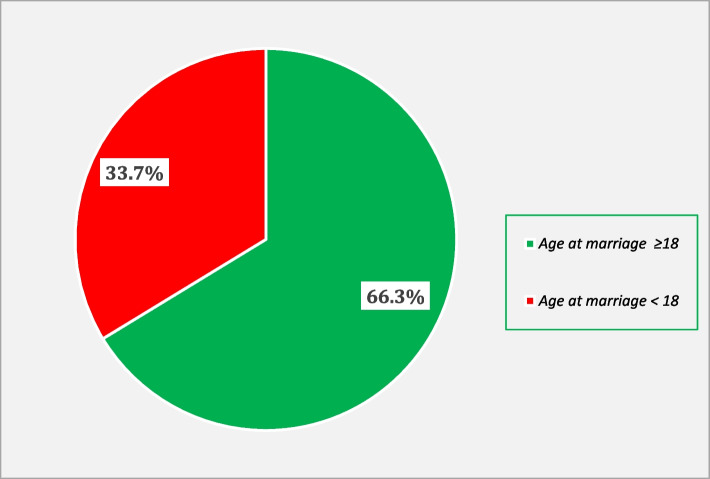
Prevalence of child marriage among reproductive age women in Harar, Ethiopia 2022. In nearly one out of every three 348 (35.29%) cases of child marriages father was the solely decision makers (Table 2).

The average age of the participants at marriage was 18.66 years old. Social norms and tradition was found to be the main cause of child marriage. According to this study, the prevalence of child marriage in Harari town was found to be 33.7% (Fig. 1).

### Factors associated with child marriage

In bivariate logistic regression analysis variables such as age, religion, women's educational level, husband's educational level, occupation of women, husband’s occupation, residence, means of engagement/marriage, consent at marriage, knowing the age of legal marriage, and decision-maker at marriage had a *p*-value less than 0.25.

In multivariable logistic regression analysis religion, women's educational level, residence, means of engagement/marriage and knowing the age of legal marriage were significantly associated with child marriage. Participants being Muslims had 2.3 times [AOR: 2.30, 95% CI (1.26, 4.19)] higher odds to practice child marriage than those whose religion was orthodox. Women who had a diploma and above educational level were 74% [AOR: 0.26 (0.10, 0.70)] less likely to practice child marriage than those who had a primary school educational level.

Women residing in rural areas had 5.39 times [AOR: 5.39, 95% CI (3.71, 7.82)] greater likelihood of experiencing child marriage than women who had been residing in urban. Those women whose marriage/engagement was arranged by others had been 2.68 times [AOR; 2.68, 95% CI (1.49, 4.82)] greater likelihood of experiencing child marriage than those who arrange their marriage by their preference. The participants who didn’t know the legal age of marriage were 4.49 times [AOR: 4.49 (2.57, 7.85)] higher odds to have been involved in child marriage than those who knew the legal age of marriage (Table 3).

**Table 2:** Marriage characteristics of participants from reproductive-age women residing in selected kebeles of Harari regional state, eastern Ethiopia, 2022, (*n* = 986)

Variables	Frequency	Percent (%)
Participant age at marriage		
≥ 18 years	654	66.33
< 18 years	332	33.67
^a^Main reason for marriage within < 18 years (*n* = 332)		
Social norms and tradition	110	33.13
To strength relationships	91	27.41
For prestige/respect	40	12.01
Difficult to get married if old	43	12.90
To have more children earlier	22	6.62
For economic purposes to get a dowry	14	4.20
To protect virginity	30	9.03
How did you engage in this marriage?		
Preference (Autonomous)	589	59.74
Arranged by others	277	28.09
Abduction	88	8.92
Unknown	32	3.25
Asked for any consent at your first marriage?		
Yes	425	43.10
No	561	56.90
Do you know the legal marriage age for girls?		
Yes	325	32.96
No	661	67.04
Legal marriage of girl's age (*n* = 325)		
≤ 15	11	3.38
16–17	76	23.38
≥ 18	238	73.23
Decision-makers for girl marriage in your locality?		
Fathers only	348	35.29
Jointly both parents	237	24.04
Community leaders	181	18.36
Religious leaders	176	17.85
^b^Others	44	4.46

^a^Indicates that multiple responses were given

^b^Indicates that (mothers only, brother, or sister)

**Table 3:** Bivariate and multivariable logistic regression analysis for factors associated with child marriage at Harari regional state, eastern Ethiopia, 2022, (*n* = 986)

Variables	Age at marriage		COR (95% CI)	AOR (95% CI)	*p*-value
	< 18 years	≥ 18 years			
Age of participant					
< 20	138	220	1.49 (1.12, 1.97)	1.08 (0.77, 1.52)	0.644
20–29	160	379	1	1	
30–39	29	45	1.53 (0.92, 2.52)	1.49 (0.83, 2.65)	0.179
> = 40	5	10	1.18 (0.40, 3.52)	2.49 (0.73, 8.54)	0.147
Religion					
Muslim	216	403	1.33 (0.96, 1.83)	2.30 (1.26, 4.19)	**0.007**
Orthodox	70	173	1	1	
Protestant	34	52	1.62 (0.97, 2.70)	1.79 (0.55, 5.84)	0.333
Other	12	26	1.14 (0.55, 2.39)	1.65 (0.16, 17.32)	0.677
Women education level					
No formal education	207	278	1.25 (0.88, 1.77)	1.80 (0.85, 3.82)	0.127
Primary school	69	116	1	1	
Secondary school	47	138	0.57 (0.37, 0.89)	1.65 (0.72, 3.79)	0.239
Diploma and above	9	122	0.12 (0.06, 0.26)	0.26 (0.10, 0.70)	**0.007**
Husband education level					
No formal education	189	227	4.22 (2.71, 6.57)	1.75 (0.90, 3.40)	0.096
Primary school	78	118	3.35 (2.05, 5.47)	1.20 (0.53, 2.70)	0.661
Secondary school	36	162	1.13 (0.66, 1.93)	0.58 (0.24, 1.38)	0.216
Diploma and above	29	147	1	1	
Occupation of women					
Housewife	219	325	2.36 (1.45, 3.83)	0.85 (0.30, 2.43)	0.761
Merchant	54	141	1.34 (0.77, 2.33)	0.47 (0.14, 1.52)	0.208
Government employee	24	84	1	1	
Others	35	104	1.18 (0.65, 2.13)	0.72 (0.25, 2.10)	0.549
Husband occupation					
Farmer	183	262	2.31 (1.52, 3.51)	0.72 (0.28, 1.84)	0.49
Merchant	81	175	1.53 (0.97, 2.42)	1.02 (0.37, 2.80)	0.968
Government employee	36	119	1	1	
Others	32	98	1.08(0.63, 1.86)	0.89 (0.33, 2.43)	0.824
Residence					
Urban	134	450	1	1	
Rural	198	204	3.26 (2.48, 4.29)	5.39 (3.71, 7.82)	**0.000**
Means of engagement					
Preference (Autonomous)	165	424	1	1	
Arranged by others	118	159	1.91 (1.42, 2.57)	2.68 (1.49, 4.82)	**0.001**
Abduction	39	49	2.05 (1.29, 3.23)	2.10 (0.74, 5.95)	0.161
Others	10	22	1.17 (0.54, 2.52)	1.41 (0.12, 16.71)	0.786
Consent was taken at marriage					
Yes	93	332	1	1	
No	239	322	2.65 (1.99, 3.52)	1.15 (0.71, 1.85)	0.581
Know the age of legal marriage					
Yes	61	264	1	1	
No	271	390	3.01 (2.19, 4.14)	4.49 (2.57, 7.85)	**0.000**
Decision-maker at marriage					
Fathers only	151	197	1.72 (1.22, 2.44)	1.20 (0.79, 1.83)	0.395
Jointly parents	73	164	1	1	
Community leaders	68	113	1.35 (0.90, 2.03)	1.97 (0.79, 4.89)	0.144
Religious leaders	34	142	0.54 (0.34, 0.86)	0.96 (0.38, 2.41)	0.929
Others	6	38	0.36 (0.14, 0.88)	0.50 (0.15, 1.72)	0.271

## Discussion

The purpose of this study was to determine the prevalence of child marriage and associated factors among reproductive-age women in the Harari region. The prevalence of child marriage was 33.7%, (95% CI: 30.8 – 36.7%) in the study area. One out of three reproductive-age females in the Harari region faced early marriage. Reproductive age women who are low in educational level, residing in a rural area, means of engagement arranged by other, Muslim religion followers were more likely to face child marriage.

The finding of our study is lower than the study conducted in Injibara town (44.8%) and in Amahara regional state (2016 EDHS analysis) (48.57%) [21, 24]. The reason for the difference may be socio-cultural differences between the two regions and time variation. Also, it is lower than the study conducted by analysis of EDHS 2016 among 20–24 aged women which were 40.3%, and a study conducted in Sub-Saharan Africa [25], it’s also lower than evidence from Mozambique (48%), and Nigeria (43%) in a similar study [26]. The disparity could be related to the study population difference, which was among those of reproductive age as against those aged 20–24 [27]. Additionally, the year of study may bring this difference, as child marriage is gradually decreasing [28]. The other thing scope of study in which the latter was done at the national level. This finding was lower than that of a Bangladeshi study (78.2%) [29]. This could be because two populations have different law enforcement of ages at marriage, religious, cultural, and social norms.

The finding of this research was higher than the research conducted on the estimation of child marriage among 20–24 girls in 2020, Ghana (19%), Zambia (29%), and Kenya (23%)[26]. The difference may be because of socio-cultural and lifestyle differences among the population. Additionally, the study was done among a specific age group, from 20–24 while our study include all reproductive-age females.

Our study revealed that religion, being Muslim had a significant association with child marriage. This result is supported by the perception that child marriage prevents girls from the western influence that leads to promiscuity and premarital pregnancy which is common/ most reflected in the Muslim community [30].

The present finding also revealed that child marriage had a significant relation to the educational level of respondents. Those who attend education to the level of diploma and above were less prone to engage in early marriage when compared to those with primary education. The finding is supported by a study done in Injibara Town, and a community-based cross-sectional study done in Sudan [21, 31]. Increasing opportunities for girls' education could be the best strategy to eliminate the practice of child marriage [32]. The higher the educational level the better they understand the consequences of child marriage on women's lives and reproductive health. In addition the more they stay in school, the more child marriage is delayed.

In this study, the rural residence was significantly associated with child marriage. When compared to their contemporaries, rural women had a 5.39 times higher chance of child marriage. This finding was supported by the study conducted in Amhara Region, Sinan District Northwest Ethiopia, and Sudan [19, 24, 31]. This may be because those living in rural areas have been far from information about the effect of child marriage on women's life. They are also prone to cultural and social norms that encourage early marriage. The other possible justification could be rural residents were low in educational achievement, which leads to poor awareness and knowledge about child marriage.

The Odds of child marriage had 2.68 times higher for those girls whose autonomy was not respected and when and to whom she has to marry was decided by others when compared to those who autonomously choose their partner. This may be justified as most family pressures were high before the age of 18 years old and they intervene in their children's life.

When compared to their contemporaries, people who do not know the legal age for marriage have a higher odd of child marriage. A study conducted in the Amahara region supports this finding [24]. This could be justified by not knowing the legal age for marriage can result in practicing child marriage.

### Strengths and limitations of the study

Even though the study used a large sample size, which enabled generalization to the source population, it was not without drawbacks. The cross-sectional nature of the study doesn’t indicate a cause-and-effect relationship. Again, there may recall bias for age at marriage since it was asked retrospectively.

## Conclusion

According to the findings of this study, the prevalence of child marriage in the study area was significantly high. The practice was more common among those who are lower in educational achievement, have rural residence, do not know the legal marital age, and whose means of engagement was decided by others. Focusing on strategies that allow intervening in these factors is helpful to end child marriage that directly and indirectly affect women's health and educational achievement.

## Data Availability

Additional data can be requested from the corresponding author upon reasonable request.
